# Proteomic Biomarkers Are Linked to QTc Interval in Patients With Chronic Heart Failure

**DOI:** 10.1002/prca.70020

**Published:** 2025-09-03

**Authors:** Mylène Barry‐Loncq de Jong, Teun B. Petersen, Sabrina Abou Kamar, Navin Suthahar, Nick van Boven, K. Martijn Akkerhuis, Peter J. van der Spek, Peter D. Katsikis, Rudolf A. de Boer, Victor A. W. M. Umans, Eric Boersma, Folkert W. Asselbergs, Jasper J. Brugts, Sing‐Chien Yap, Isabella Kardys

**Affiliations:** ^1^ Department of Cardiology Thorax Center Cardiovascular Institute Erasmus MC Rotterdam the Netherlands; ^2^ Department of Biostatistics, Erasmus MC University Medical Center Rotterdam Rotterdam the Netherlands; ^3^ Department of Pathology and Clinical Bioinformatics, Erasmus MC University Medical Center Rotterdam Rotterdam the Netherlands; ^4^ Department of Immunology, Erasmus MC University Medical Center Rotterdam Rotterdam the Netherlands; ^5^ Department of Cardiology, Northwest Clinics Alkmaar the Netherlands; ^6^ Amsterdam University Medical Centers, Department of Cardiology University of Amsterdam Amsterdam the Netherlands; ^7^ Health Data Research UK and Institute of Health Informatics University College London London UK

**Keywords:** biomarkers, heart failure, HFrEF, proteomics, QTc, QT interval

## Abstract

**Objective:**

This study investigates the link between circulating proteins and rate‐corrected QT (QTc) interval in patients with heart failure with reduced ejection fraction (HFrEF) and their association with cardiovascular outcomes.

**Methods and Results:**

We analyzed 197 HFrEF patients from the prospective Serial Biomarker Measurements and New Echocardiographic Techniques in Chronic Heart Failure Patients Result in Tailored Prediction of Prognosis (Bio‐SHiFT) study, all in sinus rhythm at baseline. Baseline QTc intervals were calculated and corrected for broad QRS complexes (>120 ms) using Bogossian's formula. Using the Somalogic‐SomaScan Assay, 1105 cardiovascular‐related proteins were measured in baseline blood samples. Linear regression identified 11 biomarkers significantly associated with QTc interval (false discovery rate [FDR] < 0.05), adjusted for age, sex, and QT‐prolonging medications. These included interleukin‐1 receptor‐like 1 (ST2) and angiopoietin‐2. An additional four biomarkers showed potential relevance (FDR < 0.1). Cox regression analysis revealed that five biomarkers—ST2, angiopoietin‐2, atrial natriuretic factor, insulin‐like growth factor‐binding protein 7 (IGFBP7), and carbonic anhydrase 4 (CA4)—were significantly associated with the composite clinical endpoint of cardiovascular death, heart transplantation, left ventricular assist device implantation, and heart failure hospitalization.

**Conclusion:**

Several cardiovascular proteins are associated with the QTc interval and adverse cardiovascular events in HFrEF patients. The observed associations highlight pathways such as inflammation, fibrosis, and angiogenesis, which may contribute to QTc prolongation and adverse outcomes in HFrEF. Further research is warranted to reveal underlying mechanisms and clinical applicability.

**Summary:**

- This study is the first to investigate the association between QTc interval and a broad panel of over 1000 plasma proteins in patients with heart failure with reduced ejection fraction (HFrEF).
- We identified 11 proteins significantly linked to QTc interval, five of which also demonstrated prognostic relevance for adverse cardiovascular outcomes.
- The associated biomarkers are linked to inflammation, fibrosis, and angiogenesis‐related pathways. These findings provide novel insights into the multifactorial mechanisms associated with QTc prolongation, potentially due to direct or indirect effects.
- The results emphasize the potential of circulating biomarkers as tools for understanding the pathophysiological processes associated with QTc prolongation and arrhythmogenesis in heart failure.
- Moreover, the identification of interleukin‐1 receptor‐like 1 (ST2), angiopoietin‐2, atrial natriuretic factor, IGFBP7, and carbonic anhydrase 4 (CA4) as shared markers of QTc interval prolongation and adverse outcomes underscores their clinical utility as both diagnostic and prognostic biomarkers.

**Abbreviations**
Bio‐SHiFT: Serial Biomarker Measurements and New Echocardiographic Techniques in Chronic Heart Failure Patients Result in Tailored Prediction of Prognosis
BNP: brain natriuretic peptide
CA4: carbonic anhydrase 4
CHF: chronic heart failure
ECG: electrocardiogram
ESC: European Society of Cardiology
HF: heart failure
HFrEF: heart failure with reduced ejection fraction
HGF: hepatocyte growth factor
HIF‐1α: hypoxia‐inducible factor 1‐alpha
HR: hazard ratio
HTx: heart transplantation
IGFBP7: insulin‐like growth factor‐binding protein 7
IPA: Ingenuity Pathway Analysis
LVAD: left ventricular assist device
LVF: left ventricular function
NT‐proBNP: N‐terminal‐pro hormone B‐type natriuretic peptide
NYHA: New York Heart Association
QTc
: rate‐corrected QT
RFU: relative fluorescent units
SIRT2: NAD‐dependent protein deacetylase sirtuin‐2
SOMAmers: Slow Off‐rate Modified Aptamers
ST2: interleukin‐1 receptor‐like 1

## 1 Introduction

Heart failure (HF) is known to be associated with multiple electrocardiographic changes. A prolonged QT or rate‐corrected QT (QTc) interval is relatively more common in patients with HF, compared to the general population, and has been linked to decreased left ventricular function (LVF) [1]. There is also some evidence that in patients with HF, a prolonged QTc interval is a risk factor for mortality, in particular sudden cardiac death and hospitalization [2]. However, this evidence is not consistently supported, and the exact role of prolonged QT interval in life‐threatening arrhythmias and sudden cardiac death, which is a relatively common cause of mortality in HF patients, has not yet been fully elucidated [3].

Although several hypotheses have been suggested, the exact mechanisms underlying the phenomenon of QT interval prolongation in HF patients remain to be clarified. Examining the circulating proteome, which encompasses proteins with putative pathophysiological functioning in various organs, could provide additional insights by identifying plasma protein profiles associated with QT interval prolongation. In previous studies, several blood biomarkers, such as brain natriuretic peptide (BNP), plasma fatty acid‐binding protein 4 (FABP4), serum albumin, hs‐CRP, and total white blood cell count, have shown to be associated with QT measures, such as QT interval and QTc [4, 5, 6]. These blood biomarkers suggested a role for a pro‐inflammatory state. BNP has also been shown to carry prognostic value for the risk of sudden cardiac death [4].

So far, these existing studies on the association between blood biomarker profiles and QT interval in HF patients are limited in number. Moreover, each of them only focused on one or two biomarkers. Insight into more elaborate protein profiles and their associations with QTc interval may contribute to a comprehensive understanding of the pathophysiological processes accompanying QT prolongation in HF patients and in its link with adverse clinical outcomes.

Therefore, the aim of this study is to investigate the association between over one thousand circulating protein biomarkers and QTc interval in a cohort of patients with HF with reduced ejection fraction (HFrEF). Moreover, the relation between proteins linked to QTc interval and adverse clinical outcomes were examined.

## 2 Methods

### 2.1 Study Population and Design

The Serial Biomarker Measurements and New Echocardiographic Techniques in Chronic Heart Failure Patients Result in Tailored Prediction of Prognosis (Bio‐SHiFT) study is a prospective cohort study of stable patients with chronic heart failure (CHF), conducted in Erasmus MC, Rotterdam, and Northwest Clinics, Alkmaar, the Netherlands. The study's design has been previously described [7]. In summary, the Bio‐SHiFT study involved the enrollment of patients aged 18 and above from the outpatient clinic who had received a diagnosis of CHF according to the European Society of Cardiology (ESC) guidelines, with a minimum CHF duration of 3 months [8]. Patients who had been hospitalized for HF in the 3 months preceding enrollment were excluded. The study followed a predetermined schedule of study visits, with appointments scheduled every 3 months. Blood samples were collected at baseline and during each follow‐up visit. The patients continued to receive routine outpatient care and treatment from their managing physicians in parallel with the study visits.

From August 2011 to January 2018, a total of 398 patients diagnosed with CHF (both HFrEF fraction and HF with preserved ejection fraction) were enrolled. For the current analysis, we focused on 382 HFrEF patients, 379 of whom had a baseline electrocardiogram (ECG) available for evaluation.

Ethical approval for the study protocol was granted by the medical ethics committee of the Erasmus MC, University Medical Center, Rotterdam, and written informed consent was obtained from all patients. The study was conducted in accordance with the Declaration of Helsinki and registered in ClinicalTrial.gov (NCT01851538).

### 2.2 Baseline Assessment and ECG Processing

Information was collected on HF‐related symptoms and New York Heart Association (NYHA) classification. A comprehensive physical examination was conducted, and information on HF etiology, cardiovascular risk factors, medical history, and treatment was retrieved primarily from hospital records. All baseline ECGs were generated utilizing the Mortara ELI350 equipment (Mortara Instrument, Inc., Milwaukee, WI, USA). Automatic analyses of the ECGs were performed using the VERITAS algorithm, which rendered assessment of the standard ECG features as used for clinical care, including QRS and QT intervals. Subsequently, all ECGs underwent manual review by a research physician. Patients who did not exhibit a sinus rhythm on the baseline ECG, including those with pacemaker rhythm and atrial fibrillation, were excluded from further analysis in this study. The QTc interval was calculated based on the automated QT intervals using Fridericia's formula, with correction applied for broad QRS complexes (equal to or exceeding 120 ms), using Bogossian's formula [9]. The Bogossian formula corrects for bundle branch block by subtracting 50% of the length of the QRS complex from the measured QT interval: QTm = QTbbb − 50% QRSbbb. An abnormal QTc was defined as a QTc duration of 450 ms or longer for men and 460 ms or longer for women [10].

### 2.3 Sample Collection and Processing

Blood samples were processed within a 2‐ho window after collection, and EDTA plasma was stored at −80°C. Proteomics measurements were performed in one batch, after completion of follow‐up. Accordingly, at the time of the outpatient visits, the results of the proteomics measurements were not available to the attending physicians. The laboratory personnel conducting the proteomics analysis was blinded for clinical data and patient outcomes. For the current analysis, proteomics measurements performed in all available baseline blood samples were used.

### 2.4 Proteomics Measurements

We employed the aptamer‐based SomaScan assay, developed by Somalogic (Boulder, Colorado, USA), to measure a comprehensive panel of plasma proteins, using 55 µL of EDTA plasma per sample, as previously described [11, 12]. SomaScan utilizes single‐stranded DNA‐based protein affinity reagents known as Slow Off‐rate Modified Aptamers (SOMAmers). SOMAmers bind proteins with high specificity and affinity, exhibiting slow dissociation rates, which helps to minimize non‐specific interactions.

The SomaScan assay results are reported in normalized relative fluorescent units (RFUs), which directly correspond to the amount of the target protein's available epitope in the original sample. Previous studies reported high assay reproducibility and low technical variability of SomaScan [13, 14]. To assess the quality of individual samples, the normalized median signal was compared with the external reference standard.

We followed Somalogic's established standard procedures for normalization, calibration, and quality control (Table S1) [13, 15]. These included that hybridization control, intraplate median signal normalization, and plate scale factors were expected to be between 0.4 and 2.5, the distribution of QC sample ratios was expected to have 85% of individual SOMAmer reagents in the total array between 0.8 and 1.2 [12, 16]. SOMAmers outside these ranges were not considered for the current study. Three hundred aptamers with non‐human or non‐validated targets, out of the total panel of 5284, were excluded from further analysis. Moreover, when multiple SOMAmer versions were present, those with the highest binding affinity were used, resulting in 4210 modified aptamers, corresponding to an equal number of proteomic biomarkers.

For the current analyses, we used a subset of 1105 proteins associated with cardiovascular diseases or cardiovascular system development and function, as determined through QIAGEN Ingenuity Pathway Analysis (IPA) [17]. We chose this approach because we aimed to explore biomarkers with potential links to QTc prolongation, thereby increasing the biological relevance of our findings. Restricting the analysis to this subset allowed for a more targeted investigation, while including non‐cardiovascular‐involved proteins may obscure relevant relations associated with cardiovascular pathophysiology. IPA uses algorithms to construct causal networks by combining biological pathways, functions, and diseases, based on relationships found in existing literature [18].

### 2.5 Clinical Study Endpoints

A clinical event committee reviewed hospital records and discharge letters to determine occurrence of the study endpoints. The primary endpoint of this study was a composite of cardiovascular death, heart transplantation (HTx), left ventricular assist device (LVAD) implantation, and hospitalization for the management of acute or worsened HF, whichever occurred first. In cases where patients reached multiple endpoints, only the first was used for primary endpoint analysis.

Hospitalization for acute or worsened HF was defined as admission to a medical facility due to an exacerbation of HF symptoms in combination with at least two of the following criteria: elevation of BNP or N‐terminal‐pro hormone B‐type natriuretic peptide (NT‐proBNP) exceeding three times the upper limit of normal, clinical signs of worsening HF such as pulmonary rales, raised jugular venous pressure or peripheral edema, increased dose or intravenous administration of diuretics, and/or the administration of positive inotropic agents [8].

### 2.6 Statistical Analysis

Continuous variables were presented as mean with SD, or median with the 25th to 75th percentile, as appropriate. Categorical variables were presented as numbers with percentages. Differences in continuous clinical characteristics between patients with QTc above or below the median and with or without sinus rhythm were assessed by Student's *t* tests or Mann–Whitney *U* tests, depending on normality of the distributions. The Chi‐squared test or Fisher's exact test, as appropriate, was used for the comparison of proportions.

Cumulative incidence of the composite endpoint was evaluated using the one minus the Kaplan–Meier estimate, while differences between patients with and without sinus rhythm were evaluated using the log‐rank test. Among patients with sinus rhythm, event‐free survival was compared between those with QTc values above versus below the median.

For assessing the relationship between QTc interval and the composite endpoint, Cox proportional hazards regression was applied. We corrected this analysis for age and sex. The proportional hazards assumption was tested using Log‐minus‐log plots and the Schoenfeld test, and no deviations were observed.

Linear regression was used to investigate the association of clinical characteristics with the QTc interval as the dependent variable. Continuous baseline characteristics were standardized (Z‐score), and categorical baseline variables were recoded into dichotomous variables based on clinically relevant categories. Assumptions of linear regression were tested by examining the distributions of residuals. The results of the linear regression analyses were visualized in a forest plot. Subsequently, multivariable linear regression was performed to investigate the association of log2‐transformed and then standardized plasma protein levels with QTc interval, while adjusting for age, sex, and the use of Class I and III anti‐arrhythmics and anti‐depressants. Multiple testing was accounted for by applying the Benjamini–Hochberg method (false discovery rate [FDR] < 0.05 and additional exploration with FDR < 0.1). Coefficients of proteins that retained a statistically significant association with the QTc interval after multivariable adjustment were visually presented in a forest plot. Subsequently, the associations of these log2‐transformed and then standardized protein levels, independently linked to QTc interval, with adverse clinical outcome were examined, using (univariable) Cox proportional hazards regression.

All data analyses were performed using R (version 4.3.1.). Statistical significance was determined at a two‐sided *p* value <0.05 for analysis of baseline characteristics and the Cox proportional hazards regression, or FDR < 0.05 and FDR < 0.1 for the proteomics analyses as described above.

## 3 Results

### 3.1 Baseline Characteristics and Clinical Endpoint

A total of 379 out of 382 patients with HFrEF had an available baseline ECG and were assessed for this study, 197 of whom had sinus rhythm at baseline (Table 1). In patients with sinus rhythm, the mean age was 61.6 ± 13.5 years, 69% were men, and 77% were in NYHA Class I–II. Mean QTc (corrected using Bogossian's formula) was 389 ± 46 ms, and median QTc (25th–75th percentile) was 392 ms (365–420).

**TABLE 1:** Baseline characteristics.

	Total	QTc below median	QTc above median	p value
Demographics	*n* = 197	*n =* 98	*n* = 99	
Age (mean [SD])	61.6 (13.5)	61.5 (13.1)	61.6 (13.9)	0.956
Male sex (%)	135 (68.5)	77 (78.6)	58 (58.6)	**0.0** **04**
Medical history				
Duration of CHF (median [IQR])	2.7[0.9,7.8]	3.4[1.3,9.8]	2.1[0.8,6.5]	**0.044**
Hypertension (%yes)	87 (44.8)	39 (40.2)	48 (49.5)	0.248
Atrial fibrillation (% yes)	31 (16.1)	11 (11.5)	20 (20.6)	0.124
Other arrhythmia (% yes)	65 (33.3)	29 (29.6)	36 (37.1)	0.336
Pacemaker (% yes)^a^	32 (16.8)	18 (18.6)	14 (14.9)	0.628
ICD (% yes)^a^	104 (52.8)	57 (58.2)	47 (47.5)	0.174
Myocardial infarction (% yes)	80 (41.2)	43 (44.3)	37 (38.1)	0.466
PCI (% yes)	72 (36.5)	39 (39.8)	33 (33.3)	0.427
CABG (% yes)	26 (13.2)	14 (14.3)	12 (12.1)	0.812
Chronic renal failure (% yes)	85 (43.6)	36 (37.1)	49 (50.0)	0.095
Diabetes mellitus (% yes)	46 (23.4)	22 (22.4)	24 (24.2)	0.897
Clinical characteristics				
BMI (mean [SD])	27.0 (4.5)	27.3 (4.6)	26.8 (4.4)	0.524
NYHA class (%)				0.348
NYHA Class I	54 (27.7)	29 (30.2)	25 (25.3)	
NYHA Class II	97 (49.7)	49 (51.0)	48 (48.5)	
NYHA Class III	43 (22.1)	17 (17.7)	26 (26.3)	
NYHA Class IV	1 (0.5)	1 (1.0)	0 (0.0)	
Systolic blood pressure (mean [SD])	117.0 (22.7)	113.9 (22.0)	119.9 (23.2)	0.069
Diastolic blood pressure (mean [SD])	70.2 (10.8)	69.3 (9.8)	71.1 (11.6)	0.257
LVEF (mean [SD])	29.1 (8.6)	28.5 (8.9)	29.8 (8.3)	0.416
Elevated jugular venous pressure (% yes)	12 (6.7)	3 (3.4)	9 (9.9)	0.073
Crackles or rales (% yes)	18 (10.1)	11 (12.4)	7 (7.8)	**0.043**
Peripheral edema (% yes)	23 (12.2)	11 (12.1)	12 (12.4)	0.138
Baseline ECG characteristics				
Heart rate (mean [SD])	66.0 (11.9)	67.6 (12.5)	64.4 (11.2)	0.062
QRS duration (median [IQR])	118.0 [105.0, 147.0]	133.5 [120.2, 154.0]	107.0 [100.0, 117.0]	**<0.001**
QT time (mean [SD])	416.4 (53.1)	400.1 (49.7)	432.6 (51.6)	**<0.001**
QTc (mean [SD])	389.3 (45.8)	354.9 (39.7)	422.5 (20.7)	**<0.001**
QTC				
Normal	191 (97.0)	98 (100.0)	93 (93.9)	
Abnormal	6 (3.0)	0 (0.0)	6 (6.1)	**0.039**
Medication use				
Beta blockers (% yes)	183 (92.9	93 (94.9)	90 (90.9)	0.417
ACE‐I (% yes)	139 (70.6)	68 (69.4)	71 (71.7)	0.840
Angiotensin 2 receptor antagonists (% yes)	53 (26.9)	29 (29.6)	24 (24.2)	0.493
Aldosterone antagonist (% yes)	151 (75.9)	76 (77.6)	73 (73.7)	0.647
Dihydropyridine calcium channel blockers (% yes)	13 (6.6)	5 (5.1)	8 (8.2)	0.566
Loop diuretics (% yes)	177 (89.8)	82 (83.7)	95 (96.0)	**0.009**
Thiazide diuretics (% yes)	2 (1.0)	2 (2.0)	0 (0.0)	0.473
Digoxin (% yes)	50 (25.5)	20 (20.4)	30 (30.6)	0.140
Anti‐arrhythmic medication (% yes)	29 (14.9)	11 (11.3)	18 (18.4)	0.239
Anti‐depressants	8 (4.1)	6 (6.2)	2 (2.0)	0.266

*Note:* A *p* value < 0.05 is considered statistically significant.

Abbreviations: ACE, angiotensin‐converting enzyme; BMI, body mass index (in kg/m^2^); CABG, coronary artery bypass graft; CHF, chronic heart failure; ECG, electrocardiogram; ICD, implantable cardioverter‐defibrillator; LVEF, left ventricular ejection fraction; NYHA, New York Heart Association; PCI, percutaneous coronary intervention.

^a^ Device categories overlap, for example, devices with a pacemaker and ICD‐function count in both categories.

Patients without sinus rhythm were significantly older (mean 65 vs. 62 years old), more often male (78% vs 69%), and had longer duration of HF (median 5.7 vs. 2.7 years) compared to those with sinus rhythm (Table S2). Over a median (25th–75th percentile) follow‐up of 25.1 (13.2–30.8) months, 35 cases of the composite endpoint occurred in patients with sinus rhythm at baseline, including seven cases of cardiovascular mortality. The cumulative incidence of the composite endpoint was higher in the patients without versus with sinus rhythm (*p* < 0.001) (Figure S1).

### 3.2 Baseline Characteristics and QTc Duration

**FIGURE 1:**
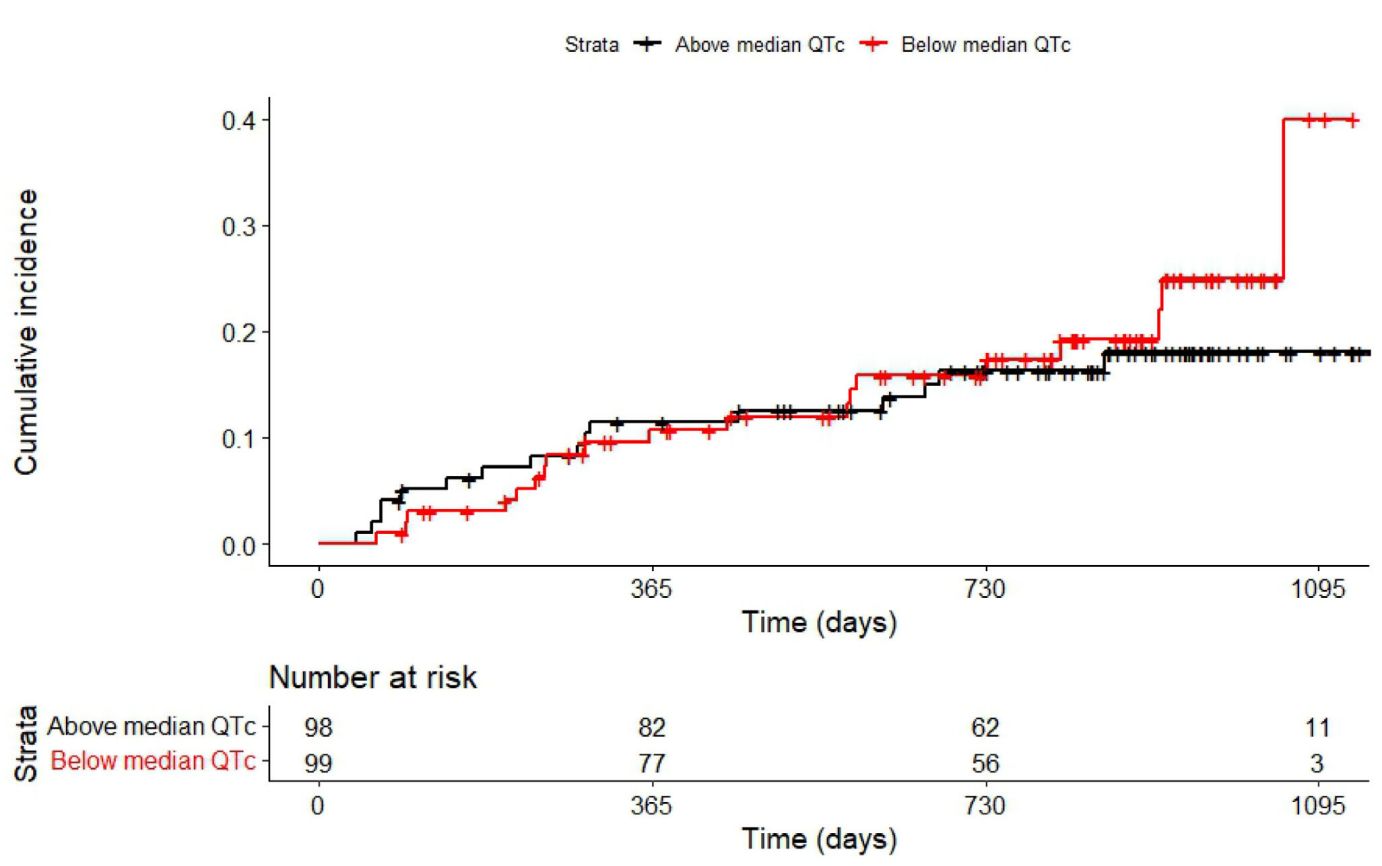
Cumulative incidence of the composite endpoint. *Legend*: Curves show a comparison between patients with a rate‐corrected QT (QTc) above and below the median.

When analyzing only the patients with sinus rhythm, patients with a QTc above the median were less often male (59% vs. 79%, *p* = 0.004) and had a shorter duration of heart failure (HF, median 2.1 vs. 3.4 years). There were no significant differences in comorbidities and symptoms, except for the occurrence of crackles or rales, which occurred more in patients with QTc below the median (12% vs. 8%, *p* = 0.043). Loop diuretics were more often used by patients with QTc above the median (96% vs 84%, *p* = 0.009), but there was no significant difference in the use of other types of medication. The cumulative incidence of the primary endpoint was similar in patients with QTc times above versus below the median (Figure 1).

**FIGURE 2:**
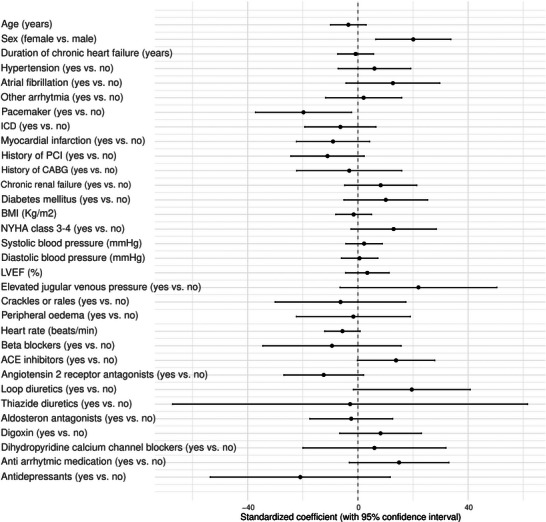
Linear regression coefficients for dichotomous‐ and continuous baseline variables in relation to rate‐corrected QT (QTc). *Legend*: Results are presented as the (standardized) beta coefficient, including the 95% confidence interval. Beta coefficients indicate the expected change in QTc (in ms) for a change of one SD (for continuous variables), or per category (for dichotomous variables). The original units of all continuous variables are presented between brackets.

The QTc interval was associated with sex, as women had a mean 20.1 ms longer QTc than men (95% CI 6.5–33.7, *p* = 0.004), which could be explained by biological differences. Moreover, the QTc interval was associated with pacemaker therapy, as QTc was 19.7 ms shorter in patients with versus without pacemaker therapy (95%CI −37.2 to −2.3, *p* = 0.026) (Figure 2), which could possibly be explained by use of Bogossian's correction formula. No significant associations were found between other variables, such as age, duration of CHF, heart rate, and the use of medication with QTc interval. Cox regression analysis, corrected for age and sex, did not show a significant association between QTc and the composite endpoint (hazard ratio [HR] per ms = 1.007, CI: 0.999–1.015, *p* = 0.111).

### 3.3 Proteomics Measurements and QTc Duration

The multivariable regression analysis, adjusted for age and sex, and corrected for multiple testing, revealed 11 proteomic biomarkers significantly associated with QTc interval (FDR < 0.05), namely metalloproteinase inhibitor 1, collectin‐11, biglycan, interleukin‐1 receptor‐like 1 (ST2), angiopoietin‐2, homeodomain‐only protein, carbonic anhydrase 4 (CA4), hypoxia‐inducible factor 1‐alpha (HIF‐1α) inhibitor, regulator of G‐protein signaling 5, hepatocyte growth factor (HGF), and NAD‐dependent protein deacetylase sirtuin‐2 (SIRT2) (Figure 3a). Four additional biomarkers were potentially relevant, with FDR < 0.1. These were angiopoietin‐related protein 3, atrial natriuretic factor, insulin‐like growth factor‐binding protein 7 (IGFBP7), and lumican. After considering medication that could prolong QT interval, regulator of G‐protein signaling 5, HGF, and SIRT2 remained significantly associated with the QTc interval (FDR < 0.05) (Figure 3b).

FIGURE 3Plasma proteins significantly associated with rate‐corrected QT (QTc). *Legend*: All coefficients are presented as milliseconds per log2‐transformed normalized relative fluorescent units (NFUs), with 95% confidence intervals. (*False discovery rate [FDR] < 0.05, other biomarkers are associated with FDR < 0.1). (a) Analyses were adjusted for age and sex. (b) Analyses were adjusted for age, sex, and QT‐prolonging medication.

**Figure:**
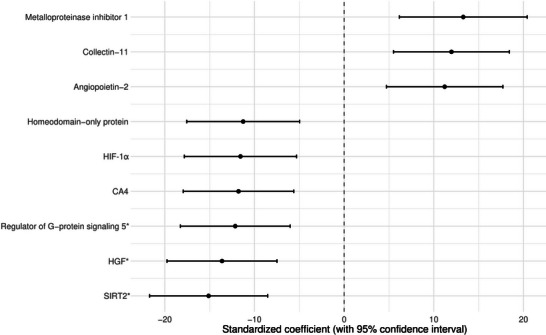

**Figure:**
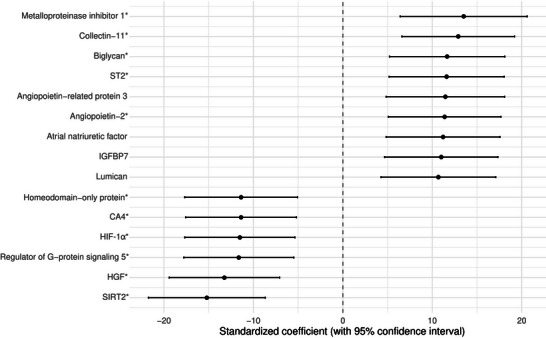

**FIGURE 4:**
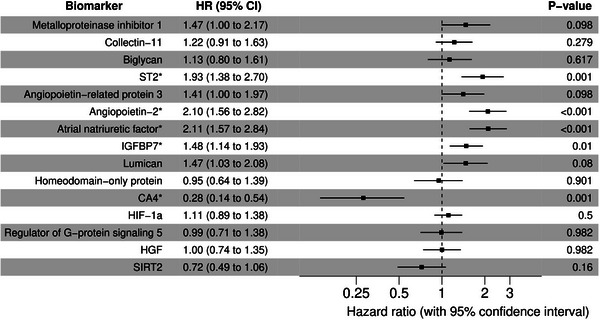
Plasma proteins that were significantly associated with rate‐corrected QT (QTc) and the composite endpoint. *Legend*: All results are presented as hazard ratios, with 95% confidence intervals. (*Biomarkers associated with the primary endpoint with false discovery rate [FDR] < 0.05).

Cox regression analyses, assessing the effects of these proteins (log2 transformed and standardized) on adverse clinical outcomes, revealed an association between ST2 (HR 1.93, 95%CI 1.38–2.70 per SD), angiopoietin‐2 (HR 2.1, 95%CI 1.56–2.82), atrial natriuretic factor (HR 2.11, 95%CI 1.57–2.84), IGFBP7 (HR 1.48, 95%CI 1.14–1.93), and CA4(HR 0.28, 95%CI 0.14–0.54) and the composite endpoint (Figure 4).

## 4 Discussion

In this study, we investigated the plasma proteome in relation to baseline QTc interval, and the association of QTc with clinical features and outcomes in patients with HFrEF. We identified 11 plasma proteins strongly associated with QTc interval.

To date, the role of QTc interval prolongation in HF remains incompletely understood. The plasma proteome provides a snapshot of the biological state and of ongoing systemic biological mechanisms; thus, it could provide additional insights by identifying plasma protein profiles associated with QT interval prolongation. However, it is important to recognize that blood biomarker expression does not necessarily provide a direct reflection of myocardial processes. It should therefore be noted that, based on this exploratory analysis, no pathophysiological conclusions can be drawn and results should be interpreted as hypotheses generating. The relationship between blood biomarker expression and complex clinical outcomes such as QTc interval prolongation is multidimensional and may be influenced by many different factors. Likely, these relationships involve complex indirect interactions between, for example, inflammatory pathways, myocardial stress response, and electrical properties of the heart.

To our knowledge, we are the first to examine the association between QTc and a broad panel over a thousand plasma proteins in HF patients. Previous studies on the associations of circulating proteins and QTc interval have only examined limited numbers of biomarkers, and research elaborating on this topic in the context of HF is scarce. In a prospective observational study in 420 patients with advanced HFrEF (excluding those with pacemakers or atrial fibrillation), a significant association was observed between higher BNP levels and QTc interval prolongation [4]. Furthermore, the combined presence of elevated BNP levels and prolonged QTc was associated with an increased risk of sudden cardiac death [4]. In a separate study, an association was identified between FABP4 and a prolonged QTc interval in patients with stable angina pectoris and chronic kidney disease [5]. Additionally, in a cohort of 1567 patients diagnosed with coronary artery disease, a prolonged QTc interval correlated with significantly lower serum albumin levels, a higher total white blood cell count, and higher levels of hs‐CRP, indicating a potential link with inflammatory processes [6]. Although not all the abovementioned biomarkers were included in our study, we did not observe an association between NT‐proBNP, FABPL, FABP, serum albumin, or CRP levels and QTc interval.

Among the 11 proteins that were strongly associated with QTc interval and the four additional proteins that showed potential relevance, several are well‐established in the pathophysiological pathways of cardiovascular diseases and prognosis of HF, such as ST2 and atrial natriuretic factor. Most of the associated biomarkers potentially implicate pathways related to inflammation, fibrosis, or alterations in the extracellular matrix.

Acute systemic inflammation is known to directly prolong the QTc interval through cytokine‐mediated effects on potassium channel expression [19]. Moreover, chronic inflammation, as seen in rheumatic diseases, is also associated with QTc prolongation [20]. Proteins found in this study that are associated with inflammation are: metalloproteinase inhibitor 1, collectin‐11, biglycan, ST2, angiopoietin‐2, atrial natriuretic factor, IGFBP7, and lumican [21, 22, 23, 24, 25, 26, 27, 28]. HGF is known for its anti‐inflammatory properties [29].

Myocardial fibrosis and age‐related changes in the extracellular matrix may also contribute to QTc interval prolongation [20, 30]. In this study, biglycan, ST2, atrial natriuretic factor, IGFBP7, and lumican are biomarkers associated with fibrotic processes [24, 26, 27, 28, 31]. Regulator of G‐protein signaling 5 is inversely correlated with fibrosis in HF and has been suggested to reduce the risk of arrhythmias [32]. Metalloproteinase‐inhibitor 1 and biglycan are biomarkers involved in processes associated with extracellular matrix composition [31, 33]. Moreover, metalloproteinase inhibitor 1 is also associated with angiogenesis and apoptosis [21, 33]. Although circulating MMP‐1 levels have not previously been associated with QTc prolongation, increased activity of MMP‐3, an upstream activator of MMP‐1, due to genetic polymorphisms, is known to be associated with a prolonged QTc interval [34].

Angiopoietin‐related protein 3 is best known for its role in lipid metabolism and is associated with the occurrence with coronary artery disease [35, 36]. However, this protein is also associated with processes involved in angiogenesis. HIF‐1α and angiopoietin‐2 are associated with angiogenesis and vascular remodeling [25, 37]. CA4 and HIF‐1α are associated with cardiac hypertrophy, whereas SIRT2 is negatively associated with hypertrophic cardiac disease [38, 39]. Homeodomain‐only protein and IGFBP7 are associated with cardiomyocyte differentiation [40, 41].

Although a pathophysiological link between the biomarkers implicated in this study, and QTc prolongation, has not yet been demonstrated in the literature, such a link could possibly be explained by the abovementioned underlying processes, through direct or indirect effects.

Moreover, four of these biomarkers, namely ST2, angiopoietin‐2, atrial natriuretic factor, and IGFBP7, were associated with a higher risk of the composite endpoint. ST2, atrial natriuretic factor, and IGFBP7 are established prognostic markers in HF [41, 42, 43]. Angiopoietin‐2 is lesser known for its prognostic role in HF, but multiple studies show an association between higher biomarker levels and adverse events [44, 45].

Several limitations of this study should be considered. Firstly, the Bio‐SHiFT study was not originally designed for the assessment of associations of plasma proteins with QTc interval, nor with arrhythmias and sudden cardiac death, both of which are relevant outcomes to this topic [2]. QT prolongation is relatively more common in HF patients, compared to the healthy population [1]. Additionally, sudden cardiac death is a relatively common cause of mortality in patients with HF. In a prospective observational registry with a median follow‐up period of 3.8 years, 48.7% of patients died, and sudden cardiac death represented 13.6% of all deaths in this population [3]. However, in our smaller cohort, a relatively low number of patients presented with abnormal QTc intervals. We corrected the QT interval for broad QRS complexes to focus primarily on true repolarization time, using Bogossian's formula [9]. Without this correction, a prolonged QT could imply a prolonged depolarization time, prolonged repolarization time, or a combination of both. This correction could have resulted in the small number of only six patients with a prolonged QTc interval (QTc duration of 450 ms or longer for men and 460 ms or longer for women). Moreover, only seven patients died of cardiovascular causes, including sudden cardiac death (3.5%). This could possibly explain why only five of the found biomarkers were associated with the clinical endpoint. Secondly, the QT interval can only be reliably measured in patients with sinus rhythm. Therefore, a relatively large portion of the original cohort had to be excluded from further analysis (48%). Patients with and without sinus rhythm significantly differed in terms of age, duration of HF, and survival, suggesting that these analyses contained a relatively healthier subgroup with less advanced disease. This difference may be because patients with more advanced HF are more prone to developing atrial fibrillation or other arrhythmias [46]. Third, we did not have access to a validation cohort that included both plasma proteomics and QTc interval measurements, nor were we able to perform additional orthogonal validation experiments. However, previous studies, including investigations in the current cohort, have shown high correlations between SomaScan assay protein measurements and immunoassay‐based measurements (Figure S2) [16, 47]. Moreover, several of our key findings are supported by consistent associations that we observed in our full cohort, which also includes patients without a baseline ECG or without sinus rhythm at baseline [16]. In this larger cohort, both ST2 and angiopoietin‐2 were also independently associated with the clinical endpoint, supporting the robustness of our results. Despite these limitations, this exploratory biomarker study offers new insights into the association between circulating cardiovascular proteins and QTc interval in HFrEF patients, proposing several biomarkers that potentially play a role in underlying direct or indirect pathophysiological pathways.

## 5 Conclusion

In conclusion, we identified several circulating cardiovascular‐related proteins associated with the QTc interval. Five of these biomarkers, ST2, angiopoietin‐2, atrial natriuretic factor, IGFBP7, and CA4, were also significantly associated with adverse clinical outcomes. Further research is necessary to fully elucidate these relationships. Nevertheless, these findings suggest that complex multifactorial systemic mechanisms are associated with QTc interval prolongation, including indirect processes linked to, for example, inflammation, angiogenesis, apoptosis, fibrosis, and the response to oxidative stress.

## Conflicts of Interest

The institution of Rudolf A. de Boer has received research grants and/or fees from AstraZeneca, Abbott, Bristol‐Myers Squibb, Cardior Pharmaceuticals GmbH, NovoNordisk, and Roche; Rudolf A. de Boer has had speaker engagements with and/or received fees from and/or served on an advisory board for Abbott, AstraZeneca, Bristol Myers Squibb, Cardior Pharmaceuticals GmbH, NovoNordisk, and Roche; Rudolf A. de Boer received travel support from Abbott, Cardior Pharmaceuticals GmbH, and NovoNordisk. Jasper J. Brugts reports an independent research grant for ISS (Abbott) paid to the Institute and has had invited speaker engagements or advisory boards with Astra Zeneca, Abbott, Bayer, Boehringer, Novartis, vifor and Zoll in the past 5 years. Sing‐Chien Yap has received honoraria from Boston Scientific, Medtronic, Biotronik, Acutus Medical, and Sanofi. In addition, he has received research grants from Medtronic, Biotronik, and Boston Scientific. Isabella Kardys has received travel reimbursement from SomaLogic and Olink. The other authors declare no conflicts of interest.

## Supporting information




**Supporting Information file 1**: prca70020‐sup‐0001‐SuppMat.docx

## Data Availability

Data used for this investigation can be obtained for the purpose of reproducing the results, upon reasonable request, from the corresponding author, in line with a data‐sharing agreement.
