# Supplementary material for: Proteomic Biomarkers Are Linked to QTc Interval in Patients With Chronic Heart Failure
Source: Proteomics Clin Appl. 2025 Sep 3;20(1):e70020. doi: 10.1002/prca.70020 (PMC12743590; doi:10.1002/prca.70020)
Supplement: Supplementary file 1 — Supporting Information file 1: prca70020‐sup‐0001‐SuppMat.docx [file PRCA-20-e70020-s001.docx]

**Supplementary Table 1: Analytic performance of the SOMAscan assay^[[1]](#footnote-1)^**

| Attribute | Metric | Criterion | Result |
| --- | --- | --- | --- |
| Precision (Total CV) | Median %  % in Tail (above 90th %) | ≤7.5%  <15% | 5.0%  11.8% |
| Accuracy | Median QC ratio  % in Tail | N/A  <15% | 1.0  2.4% |
| Signal to Noise | Median | ≥2.5 | 10.5 |
| Limit of Detection (LoD) | Median | ≤100 fM | 90fM |
| Spike and recovery | 25th–75th percentile | N/A | 85%-121% |
| Median dynamic range per SOMAmer reagent buffer | Range | N/A | 4.7 logs |
| Interfering Substances | Non-Interfering | Pass | Pass |

**Supplementary table 2:** Baseline characteristics, comparing patients with and without sinus rhythm.

**Legend**: CHF (Chronic heart failure), ICD (implantable cardioverter-defibrillator), CRT (cardiac resynchronization therapy), PCI (percutaneous coronary intervention), CABG (coronary artery bypass graft), BMI (Body mass index in kg/m^2^), NYHA (New York Heart Association), LVEF (left ventricular ejection fraction), ECG (Electrogardiogram), ACE (angiotensin-converting enzyme. A p-value <0.05 is considered statistically significant.

|  | **Total population** | **Sinus rhythm** | **No sinus rhythm** | **P-value** |
| --- | --- | --- | --- | --- |
| **Demographics** | n= 379 | n= 197 | n= 182 |  |
| Age (mean (SD)) | 63.4 (13.1) | 61.6 (13.5) | 65.3 (12.5) | **0.006** |
| Male sex (%) | 277 (73.1) | 135 (68.5) | 142 (78.0) | **0.049** |
|  |  |  |  |  |
| **Medical history** |  |  |  |  |
| Duration of CHF (median[IQR]) | 4.2[1.6,9.5] | 2.7[0.9,7.8] | 5.7[2.6,11.1] | **<0.001** |
| Hypertension (% yes) | 166 (44.3) | 87 (44.8) | 79 (43.6) | 0.897 |
| Atrial fibrillation (% yes) | 135 (36.1) | 31 (16.1) | 104 (57.5) | **<0.001** |
| Other arrhythmia (% yes) | 149 (39.6) | 65 (33.3) | 84 (46.4) | **0.008** |
| Pacemaker (% yes) | 85 (23.2) | 32 (16.8) | 53 (30.3) | **0.005** |
| ICD (% yes) | 251 (66.2) | 104 (52.8) | 147 (80.8) | **<0.001** |
| CRT (% yes) | 105 (27.8) | 0 (0.0) | 105 (57.7) | **<0.001** |
| Myocardial infarction (% yes) | 143 (38.2) | 80 (41.2) | 63 (35.0) | 0.257 |
| PCI (% yes) | 124 (32.7) | 72 (36.5) | 52 (28.6) | 0.123 |
| CABG (% yes) | 53 (14.0) | 26 (13.2) | 27 (14.8) | 0.756 |
| Chronic renal failure (% yes) | 179 (47.5) | 85 (43.6) | 94 (51.6) | 0.144 |
| Diabetes mellitus (% yes) | 98 (25.9) | 46 (23.4) | 52 (28.6) | 0.297 |
|  |  |  |  |  |
| **Clinical characteristics** |  |  |  |  |
| BMI (mean (SD)) | 27.2 (4.5) | 27.0 (4.5) | 27.4 (4.5) | 0.496 |
| NYHA class (%) |  |  |  | 0.135 |
| NYHA class I | 93 (24.7) | 54 (27.7) | 39 (21.4) |  |
| NYHA class II | 181 (48.0) | 97 (49.7) | 84 (46.2) |  |
| NYHA class III | 100 (26.5) | 43 (22.1) | 57 (31.3) |  |
| NYHA class IV | 3 (0.8) | 1 (0.5) | 2 (1.1) |  |
| Systolic blood pressure (mean (SD)) | 115.4 (21.3) | 117.0 (22.7) | 113.7 (19.6) | 0.146 |
| Diastolic blood pressure (mean (SD)) | 70.0 (10.6) | 70.2 (10.8) | 69.8 (10.4) | 0.759 |
| LVEF (mean (SD)) | 29.4 (9.6) | 29.1 (8.6) | 29.7 (10.8) | 0.666 |
| Elevated jugular venous pressure (% yes) | 25 (7.3) | 12 (6.7) | 13 (7.9) | 0.529 |
| Crackles or rales (% yes) | 45 (13.1) | 18 (10.1) | 27 (16.5) | 0.103 |
| Peripheral edema (% yes) | 45 (12.5) | 23 (12.2) | 22 (12.7) | 0.851 |
|  |  |  |  |  |
| **Baseline ECG characteristics** |  |  |  |  |
| Rhythm |  |  |  |  |
| Sinus rhythm (%) |  | 197 (100.0) |  |  |
| Atrial fibrillation (%) |  |  | 30 (16.5) |  |
| Atrial flutter (%) |  |  | 2 (1.1) |  |
| Other atrial rhythm (%) |  |  | 2 (1.1) |  |
| Pacemaker rhythm (%) |  |  | 148 (81.3) |  |
| Heartrate (mean (SD)) | 67.4 (12.3) | 66.0 (11.9) | 69.0 (12.6) | **0.018** |
| QRS duration (median[IQR]) | 140.0[110.0,173.0] | 118.0[105.0,147.0] | 165.0[138.0,188.0] | **<0.001** |
| QT time (mean (SD)) |  | 416.4 (53.1) |  |  |
| QTc (mean (SD)) |  | 389.3 (45.8) |  |  |
| QTC |  |  |  |  |
| Normal |  | 191 (97.0) |  |  |
| Abnormal |  | 6 (3.0) |  |  |
|  |  |  |  |  |
| **Medication use** |  |  |  |  |
| Betablockers (% yes) | 347 (91.8) | 183 (92.9 | 164 (90.6) | 0.534 |
| ACE-I (% yes) | 256 (67.7) | 139 (70.6) | 117 (64.6) | 0.263 |
| Angiotensin 2 receptor antagonists (% yes) | 107 (28.2) | 53 (26.9) | 54 (29.7) | 0.629 |
| Aldosterone antagonist (% yes) | 290 (76.5) | 151 (75.9) | 139 (77.2) | 0.852 |
| Dihydropyridine calciumchannel blockers (% yes) | 17 (4.5) | 13 (6.6) | 4 (2.2) | 0.067 |
| Loopdiuretics (% yes) | 350 (92.3) | 177 (89.8) | 173 (95.1) | 0.087 |
| Thiazidediuretics (% yes) | 12 (3.2) | 2 (1.0) | 10 (5.5) | **0.027** |
| Digoxin (% yes) | 126 (33.3) | 50 (25.5) | 76 (41.8) | **0.001** |
| Anti arrhytmic medication (% yes) | 81 (21.6) | 29 (14.9) | 52 (28.9) | **0.002** |
| Antidepressants | 16 (4.2) | 8 (4.1) | 8 (4.4) | 1.000 |
|  |  |  |  |  |
| **Outcomes** |  |  |  |  |
| Composite endpoint | 113 (29.8) | 35 (17.8) | 78 (42.9) | **<0.001** |
| Cardiovascular death | 33 (8.7) | 7 (3.6) | 26 (14.3) | **<0.001** |

**Supplementary figure 1:** Cumulative incidence of the composite endpoint, comparing patients with and without sinus rhythm.


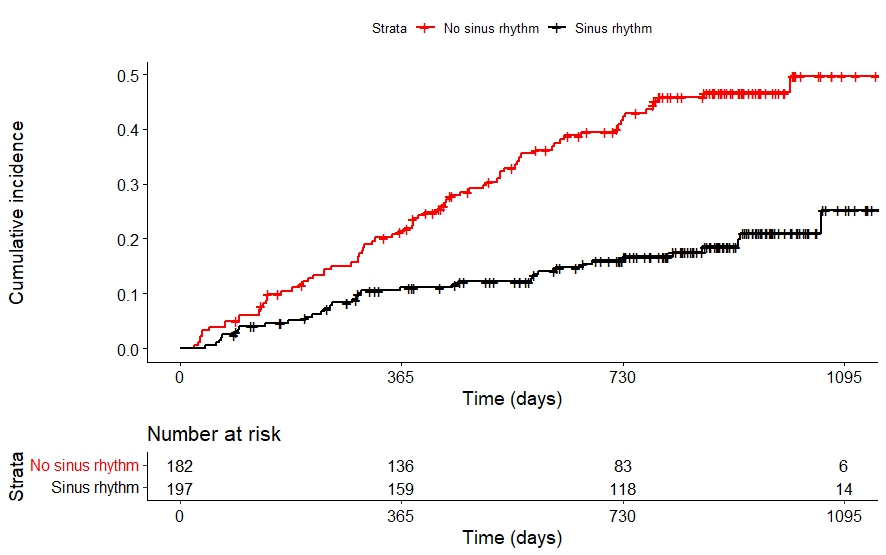


p <0.001

**Supplementary figure 2:** Available correlations of protein measurements from the SomaScan assay and clinical assays, based on previous measurements^[[2]](#footnote-2)^.


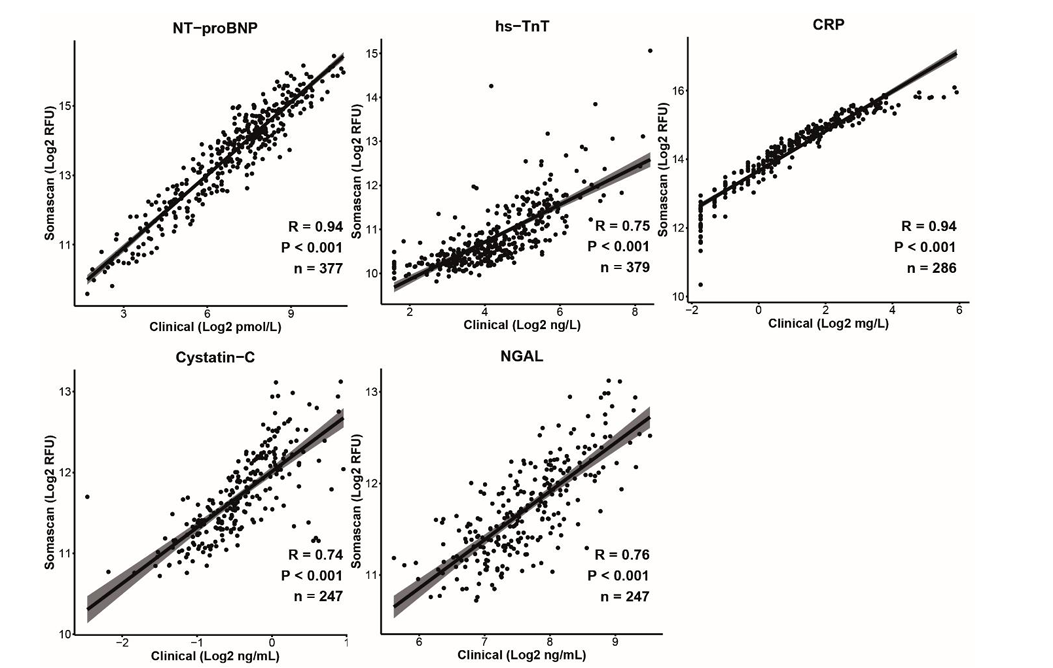


1. Petersen TB, de Bakker M, Asselbergs FW, Harakalova M, Akkerhuis KM, Brugts JJ, van Ramshorst J, Lumbers RT, Ostroff RM, Katsikis PD, van der Spek PJ, Umans VA, Boersma E, Rizopoulos D, Kardys I. HFrEF subphenotypes based on 4210 repeatedly measured circulating proteins are driven by different biological mechanisms. EBioMedicine. 2023 Jul;93:104655. doi: 10.1016/j.ebiom.2023.104655. Epub 2023 Jun 14. PMID: 37327673; PMCID: PMC10279550. [↑](#footnote-ref-1)
2. de Bakker M, Petersen TB, Rueten-Budde AJ, Akkerhuis KM, Umans VA, Brugts JJ, et al. Machine learning-based biomarker profile derived from 4210 serially measured proteins predicts clinical outcome of patients with heart failure. Eur Heart J Digit Health. 2023;4(6):444-54. [↑](#footnote-ref-2)
